# Shedding of Viral Haemorrhagic Septicaemia Virus (VHSV) from Rainbow Trout, *Oncorhynchus mykiss*, and Quantification in Waste from Processing Preclinical Fish

**DOI:** 10.1155/2023/5534720

**Published:** 2023-09-28

**Authors:** Claire Joiner, Mickael Teixeira Alves, Birgit Oidtmann

**Affiliations:** ^1^Centre for Environment, Fisheries and Aquaculture Science, Weymouth, Dorset, UK; ^2^Department for Environment, Food and Rural Affairs, London, UK

## Abstract

Viral haemorrhagic septicaemia virus (VHSV) is a fish disease notifiable to the World Organisation for Animal Health. The United Kingdom is currently free from VHSV, and the introduction and onward spread of this disease could cause major economic losses in aquaculture facilities. Legislation in Great Britain requires that imports of live fish for aquaculture purposes into declared disease-free areas are of equivalent disease-free status. However, conditions on fish products are less stringent, whereby eviscerated fish or fillets can be transported from areas with disease to areas declared disease-free. Market-size rainbow trout were experimentally infected with VHSV to investigate two important factors relevant for pathogen introduction and transmission: (1) VHSV shedding, quantified by daily assessment of viral titres in tank water samples, and (2) VHSV concentrations in liquid and solid processing waste. Evisceration and filleting preclinical fish, maceration, and wastewater separation processes within a facility were mimicked, and VHSV was quantified in each fraction of the wastewater. Shedding was detected 25 hr post-challenge. Levels increased daily to peak on day 5 post-challenge, with a calculated average titre of 1.35 × 10^3^ TCID_50_ mL^−1^ kg^−1^ fish, 1 day before clinical signs of disease. Preclinical fish contained virus levels in their kidney, skin, and muscle >10^7^ TCID_50_ g^−1^. The fish had significantly higher levels in the kidney, and evisceration led to higher VHSV concentrations in the waste compared to filleting. However, there was no significant difference in levels in wastewater released from the two processes after the removal of solids, even when macerated; average titres were >10^4^ TCID_50_ mL^−1^. The quantities of VHSV from shedding and processing can be utilised when modelling transmission and undertaking more accurate risk assessments for imports and processing of commodities, with the ultimate aim of reducing the global risk of disease from international trade and processing.

## 1. Introduction

Aquaculture is the fastest-growing global food sector and one which is set to more than double in size by 2050 [1]. Salmonids (particularly rainbow trout and salmon) are one of the most important groups of fish in aquaculture. Approximately 184,000 tonnes of salmonids, including rainbow trout (*Oncorhynchus mykiss*), brown trout (sea trout) (*Salmo trutta*), Atlantic salmon (*Salmo salar*), Arctic char (*Salvelinus alpinus*), and salmonids not elsewhere identified, were produced from aquaculture (excluding ova and juveniles) in the United Kingdom annually, averaged over the period between 2013 and 2018, with a value at approximately £800 M [2]. Global aquaculture production of rainbow trout alone reached approximately 960,000 tonnes with a value of 4,300 M USD in 2020 (FAO. 2022). Fishery and Aquaculture Statistics. Global aquaculture production 1950-2020 (FishStatJ). In: FAO Fisheries and Aquaculture Division (online). Rome. Updated 2022. (http://www.fao.org/fishery/statistics/software/fishstatj/en, accessed 08 July 2022). However, emerging diseases pose an important threat to salmonid production and could have a major economic impact on recreational angling and aquaculture businesses. Early detection and quantitative information on disease transmission for modelling disease outbreaks and undertaking risk assessments are of utmost importance in ensuring disease prevention and control.

Viral haemorrhagic septicaemia virus (VHSV) is a rhabdovirus that has been found in over 140 fish species in marine and freshwater ecosystems [3]. The virus can cause serious disease in salmonids, with one of the most susceptible species being rainbow trout (*Oncorhynchus mykiss* [4]). The European Union Reference Laboratory for Fish and Crustacean Diseases collates data on disease outbreaks in Europe, and conservative estimates indicate that 81 salmonid farms were classed as “known to be infected” for VHSV in 2019 (https://www.eurl-fish-crustacean.eu/fish/survey-and-diagnosis, accessed 13 September 2021). VHS has been reported from countries in Europe, North America, and North Asia [5]. The high mortalities, lack of available treatment, potential for spillover to novel fish species, and broad geographic distribution of VHSV, justify an integrated effort aiming to prevent and mitigate the impacts of this virus in the areas predicted at risk [3].

Movement of infected live fish is considered the most important route for the regional spread of VHSV and some other viral fish diseases between aquaculture production sites [6]. Subsequent onward transmission via water can lead to infection of naïve fish within that proximity. VHSV is primarily released horizontally through water via urine and directly from the skin from infected fish [5, 7], but to date, no data have been published on the total amount of VHSV shed into the water from infected rainbow trout. Previous studies have shown that rainbow trout that had been infected with VHSV but looked healthy had high amounts of virus in their tissues [8], but how much is released into the water from these fish while they are alive or released in wastewater from processing these fish is unknown. International trade in aquatic animal products is recognised by the World Organisation for Animal Health (WOAH) as a relevant route of trans boundary pathogen spread [9]. Where aquatic animals are further processed in a given importing country, both solid and liquid waste may be generated that could carry relevant pathogen load and lead to pathogen introduction and onward spread [6, 8, 10–17]. This raises important questions concerning the transmission of VHSV from clinically healthy-looking rainbow trout.

Current legislation in Great Britain (GB), in the form of retained EU Commission Regulation 1251/2008 [18] (amended by The Animals, Aquatic Animal Health, Invasive Alien Species, Plant Propagating Material and Seeds (Amendment) (EU Exit) Regulations 2020 [19], and The Official Controls (Animals, Feed and Food, Plant Health Fees, etc.) Regulations 2019 [20], as well as the respective GB Aquatic Animal Health Regulations 2009 [21–23]), minimises the risk of notifiable disease transmission by only allowing live fish to be imported from sources with equivalent or higher health status to the destination. Trade in eviscerated fish carcases imported into GB or traded within is not restricted on fish health grounds. Therefore, eviscerated fish carcases can be imported from sources that are not recognised as VHS free. Many countries and trading areas operate similar fish health regimes, including the EU, in accordance with the Animal Health Law, Regulation (EU) 2016/429 [24].

The WOAH recommends that countries free of a specified pathogen should assess the risk of introduction associated with trade in aquatic animal products not listed as safe for trade. Based on risk assessments, an importing country can manage the risk to an acceptable level—where possible by applying post-import measures [9]. The different risk levels of commodities are reflected in the WOAH Aquatic Animal Health Code, which aims to assure the sanitary safety of international trade in aquatic animals and their products [9]. Most aquatic animal risk assessments have dealt with live animals [25], but both Australia and New Zealand have assessed the risk of disease introduction through the importation of salmonid carcases [26, 27], and a qualitative import risk assessment was undertaken to assess the likelihood of introduction and establishment of VHSV geno group Ia in England and Wales, via the processing of imported rainbow trout carcases from continental Europe [14]. The necessary steps and associated risk pathways for VHSV introduction and exposure were identified, and concluded a high likelihood that (1) a consignment will contain at least one infected fish, (2) VHSV survives transport to the United Kingdom, (3) VHSV survives processing, (4) solid and liquid waste is produced, (5) virus is released into water, (6) the discharge of liquid waste goes to mains drainage, (7) virus is not inactivated by treatment, and (8) a susceptible individual will be exposed. Data to support quantitative risk assessments of pathogen introduction via import of products are limited, and a more accurate assessment of the risks associated with processing can be achieved by taking a more quantitative approach.

The aims of this study were to (1) quantify the shedding rates of VHSV during infection, (2) quantify VHSV released during processing (eviscerating and filleting) of clinically healthy VSHV-infected fish, (3) put virus released via liquid waste into context with viral load in fish tissues. This data will support quantitative risk assessments of pathogen transmission and introduction via the import of products in the United Kingdom and globally.

## 2. Materials and Methods

### 2.1. Virus Culture and Quantification by Titration

VHSV J167 genogroup Ia [28] was propagated on *Epithelioma papulosum cyprini* (EPC) cells [29] (ATCC® CRL-2872™) originating from common carp *Cyprinus carpio*, but shown to be contaminated with cells from fathead minnow *Pimephalespromelas* [30] at 15°C, in Glasgow's minimum essential medium (GMEM) supplemented with 10% foetal bovine serum (FBS), 1% L-glutamine (200 mM solution), 0.16% sterile tris solution (120 g L^−1^), 0.5% sterile 7.5% sodium bicarbonate and 1% penicillin–streptomycin.

All tissue samples were incubated at 4°C at a 1 in 10 dilution (w/v) in GMEM, supplemented as above and with 1% antibiotic antimycotic (100x) solution. The following day, the tissues were ground, re-suspended in the medium, centrifuged at 2,700 × g for 15 min at 4°C and the supernatant titrated.

### 2.2. Titration

Virus levels were quantified by end-point dilution assays. Serial 10-fold dilutions were performed on EPC cells in 96-well plates with dilutions from 10^−1^ to 10^−8^, six replicates per dilution and incubation at 15°C for 7 days. The 50% tissue culture infectious dose (TCID_50_) was calculated [31]. For tissue samples, the starting material was virus contained in 1 g of tissue homogenised in 9 mL media, then 0.02 mL of this was added to 0.18 mL GMEM per well for the first dilution in the titration-equivalent to 0.002 g in a total volume of 0.2 mL. After mixing, one in ten of the mixture was used to make the next log_10_ dilution (0.02 mL and 0.0002 g removed), leaving 0.18 mL and 0.0018 g per well. We calculated the TCID_50_ value per volume of well; therefore, the TCID_50_ was given per 0.18 mL and also per 0.0018 g. The virus titre per ml was then obtained from the reciprocal of the TCID_50_ value, corrected for the amount of virus used to infect one well. The TCID_50_ mL^−1^of the sample was calculated by multiplying by (1/0.18 mL), and in the same fashion the TCID_50_ g^−1^of the sample could be calculated by multiplying by (1/0.0018 g) (which is equivalent to multiplying the TCID_50_ mL^−1^ by 100). Without prior dilution, 0.02 mL of the tank water samples and supernatant (from the wastewater samples from gutting and filleting fish) was added to 0.18 mL GMEM per well for the first one in tendilution of the titration—in this case, the concentration of the virus is calculated as the TCID_50_ mL^−1^.

TCID_50_ values are also presented normalised to the weight of the solid fraction obtained after centrifugation of the liquid waste. For this purpose, the TCID_50_ in the total volume of the liquid fraction of the waste (1,000 mL) was divided by the solid fraction weight. Representing the data in this way facilitates analysing whether the quantity of tissue or blood in the liquid waste had any impact on the concentration of virus per ml of liquid waste.

### 2.3. Exposure of Rainbow Trout to VHSV

Rainbow trout (triploid females) were reared in Cefas' specific-pathogen-free (SPF) facilities from SPF ova at 8°C. Once reaching market size (average weight 326 g and average standard length 26.4 cm (measured to the caudal peduncle), the temperature was increased by 1°C a day to 10°C. Five groups of 18 rainbow trout were moved to 300 L (stocking density 22 kg m^−3^), continual flow, cylindrical freshwater tanks also at 10°C. The fish were starved for approximately 15 hr prior to movement. The fish were given at least 5 days to acclimate to the new tanks, until staggering the viral exposure to each tank. The fish were not fed the morning of the exposure. Temperature, oxygen (>7 mg L^−1^), and flow rates (3 L min^−1^) were measured daily, lighting was approximately 150-250 lux at the water surface for a day length of 16 hr (with 30 min gradual increase and decrease), and the fish were fed sinking 4.5 mm trout pellets three times a day (0.6%–0.7% bodyweight day^−1^).

Four groups of 18 rainbow trout were exposed in their tanks to VHSV J167 at 1 × 10^5^ TCID_50_ mL^−1^ by static immersion for 4 hr. Water was returned to the tanks after the exposure period. Exposure of the experimental groups was staggered with weekly intervals, so that sampling and follow-up processing could be managed logistically. It was important to ensure samples could be obtained at the preclinical stage. A negative control group of 18 rainbow trout was mock-challenged with GMEM for 4 hr and ran throughout the study.

### 2.4. Water Samples from Experimental Tanks (Shedding of VHSV)

**Figure 1:**
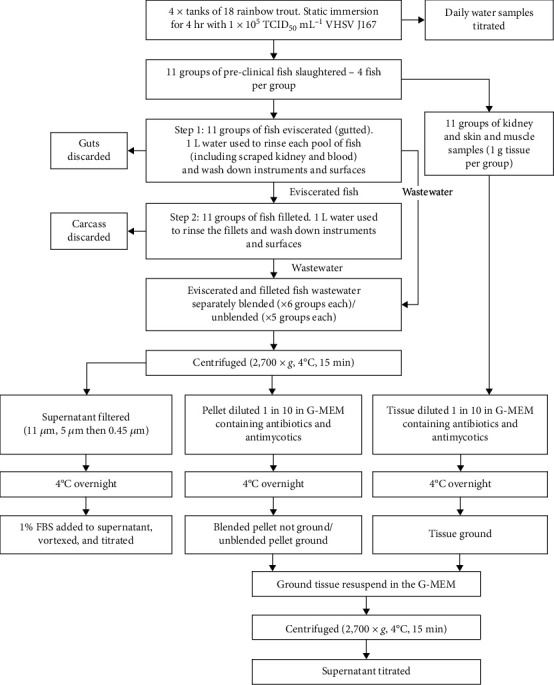
Flow diagram summarising the methods.

An overview of the experimental setup is shown in Figure 1.

Two 50 mL water samples were taken from each tank at 10 min and 4 hr during exposure from the top and bottom half of the tank and titrated. Water samples were then taken daily throughout the study, with a continuous flow of 3 L min^−1^. About 50 mL samples were taken from four areas of the tank, on opposite sides at the top half of the tank and opposite sides at the bottom half of the tank and titrated.

### 2.5. Collection of Wastewater from Eviscerating (Gutting) and Filleting Fish (Figure 1)

After first clinical signs were observed in at least one fish in a given challenge tank, fish that were not displaying clinical signs were slaughtered in groups of four (from the same tank). Fish were sampled before feeding on day 6 or 8 post-challenge (pc), mimicking procedures of manuals laughter of fish followed by evisceration and filleting. Slaughter took place by concussion of the brain followed by exsanguination by removal of the gills. The processing took place in two steps following slaughter: (1) removal of guts and (2) preparation of muscle fillets (skin on). Gills and internal organs from step 1 and the remaining carcase after filleting in step 2 were discarded, blood were retained. For each step, a pre-measured volume of 1 L non-chlorinated tap water (at room temperature) was used to wash the fish carcases (step 1) or muscle fillets (step 2), and any instruments and surfaces that had come into contact with the fish during this process and was retained. Clean instruments and a clean surface tray were used for step 2. Using this process, a total of 11 pools of water samples were generated from each step.

Step 1: Collection of wastewater from eviscerating fish. Four fish were manually gutted (sequentially) on a clean stainless-steel tray and guts were discarded. About 250 mg of kidney per fish was sampled. Virus was quantified by titration (described above) from 1 g of pooled tissue (from each group of four fish). The 1 L tap water was used to (a) rinse the eviscerated fish while scraping the remaining kidney from the carcase, cleaning the body cavity from kidney tissue and blood, then (b) lightly wash the carcases (including the heads) in a bucket, and (c) rinse the tray and instruments. The liquid waste generated from these processes was pooled, bringing it back close to the initial volume of 1 l.
Step 2: Collection of wastewater from filleting fish. The four eviscerated carcases were filleted on a clean stainless-steel tray. About 250 mg of muscle, with skin attached (roughly cube shape), per fish was sampled from the centre of the fillet. Virus was quantified by titration (described above) from 1 g of pooled tissue (from each group of four fish). The 1 L tap water was used to (a) rinse the fillets in a bucket, discarding the fillets after rinsing and (b) rinse the tray and instruments. The liquid waste was pooled, again, bringing it back close to the initial volume of 1 L.

### 2.6. Further Processing of Liquid Wastewater Samples

To mimic maceration at a processing facility, six of the water samples from the eviscerated and filleted fish were immediately blended in a 1 L stainless steel blender (Waring, 21800ESK), three periods of 10 s; five of the water samples were not blended. All liquid waste samples were then centrifuged at 2,700 × g for 15 min at 4°C. Virus was quantified in both the solid (pellet) and liquid (supernatant) fractions of the sample. The liquid fraction was vacuum filtered to remove potential contaminants, first through an 11 *µ*m nylon net filter, followed by a 5 *µ*m cellulose nitrate membrane filter, then through a 0.45 *µ*m polyethersulfone membrane filter. The filtrates were stored at 4°C overnight. The following day, 0.1% FBS was added to the filtrates and titrations were performed.

To assess if filtering and overnight storage might influence virus titres measured in tissue culture, ten replicates of fresh tank water spiked with VHSV at 1 × 10^5^ TCID_50_ mL^−1^ were titrated without filtering and when filtered as described for the wastewater samples. This was performed immediately and compared to overnight incubation at 4°C.

The solid fraction from the wastewater samples was re-suspended at a one-in-ten dilution in GMEM, supplemented as above and with 1% antibiotic-antimycotic (100x) solution and also stored at 4°C overnight before titrating the following day. The solid fraction from unblended water samples was ground with sand before centrifugation and titration.

### 2.7. Near-Moribund Fish, Mortalities, and Survivors

About 1 g kidney and 1 g muscle, with skin attached, were taken from all fish developing physical clinical signs of disease during the study period, near-moribund fish, and any mortalities. The experiment was terminated on day 8 post-challenge. Samples were also taken from all survivors at this point and five negative control fish. These samples were frozen at −70°C, and the virus was quantified by titration (described above) within 4 months.

### 2.8. Ethical Statement

The authors confirm that the ethical policies of the journal, as noted on the journal's author guidelines page, have been adhered to and Cefas Animal Welfare and Ethical Review Body approval was granted. The study conformed to UK legislation under the Animals (Scientific Procedures) Act 1986 Amendment Regulations (SI 2012/3039) for the care and use of laboratory animals.

### 2.9. Statistics

Differences in tissue titre between fish states (mortality, near-moribund, or day 8 survivors (with or without internal signs of disease)) were analysed with linear regressions. Paired *t*-test was used to compare muscle with skin attached and kidney titres. The paired *t*-test was applied to all states combined and to each fish state. A multilevel linear regression was used to estimate the effects of the type of processing (eviscerated or filleted), the blended status (blended or unblended) and the sample day (6 or 8) on the tissue titre (skin and muscle or kidney) and on the wastewater supernatant titre. The sample day was assumed to be nested within the blended status, the latter being nested in the type of processing. The model containing all data levels (type of processing, blended status, sample day) was compared to the reduced model with no sample day level, which was compared to the reduced model with no blended level. In addition, paired *t*-tests were used to compare the titre in tissue and wastewater for all types of tissue, for skin and muscle tissue, and for kidney tissue. Prior to the statistical analysis, titres were log-transformed to satisfy data normality assumptions of the *t*-test and residual normality in linear regressions. All statistical analyses were performed using the *R* statistical software [32].

## 3. Results

### 3.1. Water Samples from Exposure Tanks—VHSV Shedding (Figure 2)

Water samples (at 10 min and 4 hr) to measure VHSV exposure levels during the challenge showed titres remained at 1 × 10^5^ TCID_50_ mL^−1^ in each tank with a maximum decrease of 0.59 logs_10_ (data not shown). Samples taken from the four areas of the tank (aerated with continuous flow), at a single time point throughout the challenge, were within 1 log_10_ of each other (data not shown). VHSV shedding was detected 25 hr pc, in one exposure tank (at levels close to the limit of detection of the assay, 1.76 × 10^1^ TCID_50_ mL^−1^) and was calculated to be 3.00 TCID_50_ mL^−1^ kg^−1^ fish (average log_10_ titresacross all four tanks = 0.52 TCID_50_ mL^−1^ kg^−1^). Levels increased daily from this point to peak at 118 ± 3 hr (day 5) pc with a calculated average titre across all four tanks of 1.35 × 10^3^ TCID_50_ mL^−1^kg^−1^ fish (average log_10_ titres = 3.17 TCID_50_ mL^−1^ kg^−1^), this was before any clinical signs of disease apart from a slight decrease in appetite. At 142 ± 4 hr (day 6) pc, virus levels decreased to a calculated average of 1.08 × 10^2^ TCID_50_ mL^−1^kg^−1^ fish (average log_10_ titres = 1.93 TCID_50_ mL^−1^ kg^−1^) across all four tanks. Signs of disease had increased and included one near-moribund fish, which was removed and sampled after the water sample was taken. Removal of fish showing no clinical signs of disease from the challenge tanks in groups of four for slaughter took place afterwards. Virus was still detected in one of two exposure tanks at 186 ± 1 hr (day 8) pc but at levels close to the limit of detection of the assay.

### 3.2. Wastewater from Processing (Figure 3 and Supplementary 1)

All spiked samples, after filtering and storing at 4°C overnight, had titres within 0.5 log_10_ of the spiked samples titrated immediately (data not shown). Therefore, it was considered that there was full recovery of virus and no effect from filtering and overnight storage. No statistical difference was found between the wastewater titre in waste between day 6 and day 8 processing (liquid waste ml^−1^: *F* (4,20) = 0.58, *p*=0.68; liquid waste normalised g^−1^ solid: *F* (4,20) = 0.52, *p*=0.72), and between blended and unblended samples (liquid waste mL^−1^: *F* (2,20) = 0.76, *p*=0.49; liquid waste normalised g^−1^ solid: *F* (2,20) = 2.03, *p*=0.17), therefore this data was combined for further analyses.

The wastewater produced from both eviscerated and filleted preclinical fish contained infectious levels of virus in the liquid and solid fractions of the wastewater. No statistical difference was found between VHSV levels in the wastewater between eviscerating fish and filleting fish for either liquid waste titre mL^−1^ (*β* = 0.14, *t* (20) = 0.28, *p*=0.78) or liquid waste normalised g^−1^ solid (*β* = −0.15, *t* (20) = −0.28, *p*=0.78); however, the variation was greater when eviscerating fish: average virus titres (*n* = 11 pools of four fish) in the liquid fraction of the wastewater from eviscerated fish were 8.01 × 10^5^ TCID_50_ mL^−1^ (average of log_10_ values = 4.41 TCID_50_ mL^−1^, SD = 1.53) and filleting fish 3.39 × 10^4^ TCID_50_ mL^−1^ (average of log_10_ values = 4.27 TCID_50_ mL^−1^, SD = 0.58). Average virus titres (*n* = 11 pools of four fish) in the solid fraction of the wastewater from eviscerated fish were 2.63 × 10^9^ TCID_50_ g^−1^ (average of log_10_ values = 8.33 TCID_50_ mL^−1^, SD = 1.41) and filleting fish 3.65 × 10^7^ TCID_50_ g^−1^ (average of log_10_ values = 7.38 TCID_50_ mL^−1^, SD = 0.39). VHSV titres were significantly lower in the wastewater than in the tissues (*m* = −2.59, *t* (21) = 6.71, *p* = 1.23 × 10^−6^). The mean difference in kidney tissue compared to wastewater (*m* = 3.97, *t* (10) = 8.35, *p* = 8.07 × 10^−6^) was higher than the mean of the differences in skin and muscle compared to wastewater (*m* = 1.20, *t* (10) = 10.09, *p* = 1.47 × 10^−6^).

### 3.3. VHSV Levels in Processed Preclinical Fish Samples (Figure 3 and Supplementary 1)

Average VHSV titres (*n* = 11) in the kidney of eviscerated and filleted fish were 1.97 × 10^10^ TCID_50_ g^−1^ (average of log_10_ values = 9.38 TCID_50_ g^−1^, SD = 0.97) and in the skin and muscle tissue samples were 1.11 × 10^7^ TCID_50_ g^−1^ (average of log_10_ values = 6.47 TCID_50_ g^−1^, SD of log_10_ values = 0.74). VHSV titres were statistically significantly higher in the kidney than in the skin and muscle (*β* = 2.91, *t* (20) = 7.92, *p* = 1.37 × 10^−7^). The unblended wastewater produced from eviscerating fish contained a larger amount of solid than when blended, with on average 13.11 g if unblended (*n* = 5) compared to 3.34 g if blended (*n* = 6). The same was observed for filleted fish, with on average 4.01 g if unblended (*n* = 5) compared to 2.75 g if blended (*n* = 6).

### 3.4. VHSV Levels in Near-Moribund Fish (Figure 4 and Supplementary 2)

Near-moribund fish typically had darker skin, were lethargic, with loss of appetite, some with visible exophthalmia and often drifting at the water's surface. They were first observed, removed, and sampled on days 6, 7, and 8 pc in the different exposure tanks. There were 10 near-moribund fish in total. Average titres in the kidney were 4.15 × 10^10^ TCID_50_ g^−1^ (average of log_10_ values = 10.26 TCID_50_ g^−1^, SD = 0.81); average VHSV titres in the skin and muscle were 4.94 × 10^7^ TCID_50_ g^−1^ (average of log_10_ values = 7.38 TCID_50_ g^−1^, SD = 0.72). Two of the 10 showed no internal signs of disease yet still had relatively high levels of virus in the skin, muscle, and kidney samples. The highest titre of virus in both the kidney and the skin and muscle was in a near-moribund fish with no internal signs of disease, titres were 1.2 × 10^11^ and 1.2 × 10^8^ TCID_50_ g^−1^ in the kidney and the skin and muscle, respectively.

### 3.5. VHSV Levels in Mortalities (Figure 4 and Supplementary 2)

Three mortalities occurred overnight on days 7 and 8 pc, and all showed signs of VHS in the post-mortem. Average VHSV titres in the kidney were 2.04 × 10^10^ TCID_50_ g^−1^ (average of log_10_ values = 9.41 TCID_50_ g^−1^, SD = 1.53); average titres in the skin and muscle were 2.87 × 10^7^ TCID_50_ g^−1^ (average of log_10_ values = 6.8 TCID_50_ g^−1^, SD = 1.02). All had internal signs of disease and showed haemorrhaging in the muscle tissues and adipose tissue surrounding the gut.

### 3.6. VHSV Levels in Survivors (Figure 4 and Supplementary 2)

Final samples were taken day 8 pc for processing (eviscerating and filleting). The remaining 15 fish were classed as survivors. None of the survivors had external signs of disease, and 11 of the 15 survivors also showed no internal signs of disease. These survivors had viral titres statistically significantly lower than viral titres of near-moribund fish in the kidney (*β* = −3.21, *t* (23) = −5.37, *p* = 1.88 ×10^−5^) and skin and muscle (*β* = −2.18, *t* (23) = −7.06, *p* = 3.42 × 10^−7^). However, even fish without internal signs of the disease reached relatively high titres, particularly in the kidney. Average VHSV titres in the kidney were 9.24 × 10^8^ TCID_50_ g^−1^ (average of log_10_ values = 7.06 TCID_50_ g^−1^, SD = 1.76); average titres in the skin and muscle were 5.76 × 10^5^ TCID_50_ g^−1^ (average of log_10_ values = 5.2 TCID_50_ g^−1^, SD = 0.78).

### 3.7. VHSV Levels in Combined Fish Tissues (Supplementary 1 and Supplementary 2)

**Figure 3:**
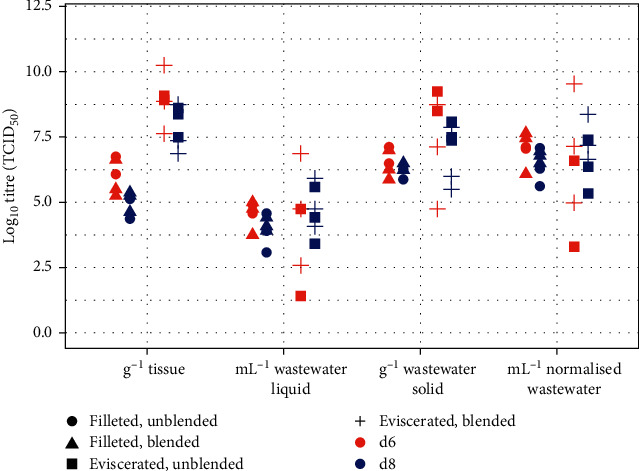
Scatterplot of log_10_ VHSV titres (TCID_50_) in wastewater from the slaughter of preclinical rainbow trout challenged with VHSV. One data point represents the titre from a group of four fish. Titres are shown g^−1^ fish tissue, mL^−1^ of the liquid fraction of the wastewater, mL^−1^of the solid fraction of the wastewater and mL^−1^ normalised wastewater (the TCID_50_ mL^−1^ in the liquid fraction multiplied by the total volume of wastewater (1,000 mL) divided by the weight of the solid fraction).

**Figure 4:**
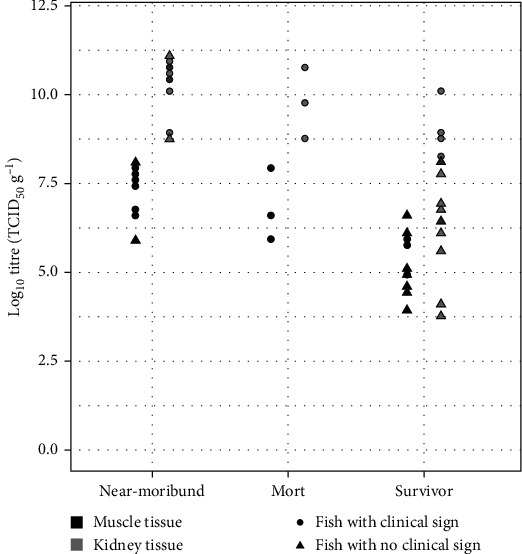
Scatterplot of log_10_ VHSV titres (TCID_50_ g^−1^) in the skin and muscle and the kidney for near-moribund fish, mortalities, and day 8 survivors. When referring to fish with no clinical signs, near-moribund fish showed no internal clinical signs of disease, and survivors showed no external or internal signs except a group decrease in appetite.

Virus was not detected in any of the control fish (data not shown), but all fish that had been subject to the immersion challenge had become infected (Figures 3 and 4). VHSV titres in kidney tissue were statistically significantly higher than in skin and muscle at the 5% significance level in all the challenged fish combined (*m* = 2.34, *t* (27) = −9.11, *p* = 1.02 × 10^−09^). VHSV titres were also statistically significantly higher in the kidney compared to the muscle in mortalities (*m* = 2.95, *t* (2) = −26.48, *p*=0.0014), near-moribund fish (*m* = 2.88, *t* (9) = −15.22, *p* = 1 × 10^−07^) and day 8 survivors (*m* = 3.42, *t* (10) = −7.45, *p* = 5 × 10^−03^). The least significant difference between the tissues was in day 8 survivors, at the point of study termination, that had no internal signs of disease (*m* = 1.29, *t* (3) = −2.80, *p*=0.0187).

## 4. Discussion

This study aimed to fill some of the current data gaps specific to VHSV shedding and VHSV in solid and liquid waste produced from processing rainbow trout. This was achieved by replicating the infection, slaughter, gutting, and filleting processes. The measurements of VHSV titres in the tank water allowed quantification of shedding throughout the disease progression. The rainbow trout shed from as early as 25 hr and peaked before signs of disease. Pre-clinical fish that were slaughtered had relatively high levels of virus in the skin and muscle sampled. The significantly higher levels in the kidney led to higher levels in the wastewater from eviscerating fish, compared to filleting, due to the solid fraction that is discarded in the wastewater during processing. However, after the removal of the solid fraction in the wastewater, there was no significant difference between the two processes, even if the wastewater was macerated prior to removing the solid fraction.

### 4.1. Shedding

The first detection of VHSV (geno group Ia) from preclinical challenged fish in the tank water, at 25 hr pc, is similar to that found from 19 g rainbow trout (Cefas, unpublished data). However, the virus strain, dose, species, size, or age of the fish may play a role in the speed of transmission. Specifically, VHSV geno group IV a was previously detected in 1-year-old Pacific herring (*Clupea pallasii*) at 4–5 days [33], and VHSV geno group IVb in juvenile muskellunge (*Esox masquinongy*) was first detected at 3–5 days (depending on initial exposure) [34].

The shedding in this study increased from day 1 to peak at day 5 with levels that are likely to infect susceptible species. Epizootics was found to be readily initiated in naïve stocks of market-size rainbow trout after a 4-hour immersion in 1 TCID_50_ mL^−1^ VHSV (https://sciencesearch.defra.gov.uk/ProjectDetails?ProjectId=16429, accessed 6 July 2023). Peak shedding before any clear signs of disease and preceding mortality has also been the case for VHSV Iain 19 g rainbow trout (Cefas, unpublished data) and VHSV IVa in Pacific herring [33]. The ability to detect the virus at this early stage via a non-invasive method infers that early detection and control measures could take place, limiting further transmission. However, until the disease is recognised and in the case of emergency harvest, fish will continue to be processed in the preclinical stage of infection if appearing healthy and, if eviscerated, can be placed on the market and processed in the United Kingdom and any of the EU member states [18, 21–24, 35].

### 4.2. VHSV Levels in Pre-Clinical Fish

The relatively high levels of virus in the kidney of pre-clinical fish, in the solid fraction of the wastewater from eviscerating pre-clinical fish, in the skin and muscle of pre-clinical fish, and in the solid fraction of wastewater from filleting pre-clinical fish were consistent with high levels previously published by Oidtmann et al. [8] in a liver, spleen and head-kidney pool (avg. 1.95 × 10^10^ TCID_50_ g^−1^) and in muscle (4.17 × 10^7^ TCID_50_ g^−1^). The results confirm that the tissues of fish, that appear visually free of disease, can in fact pose a potential high risk for disease transmission and introduction to disease-free areas. Titres of virus in the kidney were statistically significantly higher than in the skin and muscle. The smallest difference was in survivors without clinical signs and has previously been described [8]. It is likely due to the stage of infection in the fish, with the infection starting in the skin and muscle. The fish sampled for processing may have all been at a later stage of disease development than the survivors, even though they displayed no internal clinical signs, as they all had a higher statistical difference in levels of virus in the kidney compared to the skin and muscle.

### 4.3. Risk of Imports of Pre-Clinical Fish for Processing and Wastewater Produced

Historically, the import of aquatic animal products for human consumption—even from farms infected with exotic pathogens—was assumed to carry a low risk of disease introduction [36, 37]. It has since been suggested that processed fish from an infected population and effluent from the processing facilities could pose a significant risk for spreading virus [6, 8, 10–17]: (a) Vennerström et al. [38] detected virus or viral RNA in sea water and in liquid waste from processing plants, so concluded that it is possible that processing plants handling VHSV-positive fish and the surrounding environment can become contaminated with the virus; (b) the spread of the disease infectious salmon anaemia (ISA) appears to have been associated with harvesting in both Scotland and Norway [39]; (c) there is evidence that sleeping disease was introduced into a UK fish farm via rainbow trout carcases from Europe [40, 41]; (d) the most likely cause of a case of VHSV on a Swiss farm with a processing facility was the import of trout carcases for processing [42]; and (e) the outbreak of VHS in a rainbow trout farm in the United Kingdom in 2006 highlighted processing of aquatic animal products as one of the potential pathways of introduction of exotic pathogens [28]. The quantities of virus in the wastewater produced in this study, from eviscerating and filleting the pre-clinical fish, were lower than in the tissues but high enough to support the suggestions that processed fish from an infected population and effluent from the processing plant could pose a significant risk for transmission.

### 4.4. Factors Influencing Introduction and Spread

**Figure 5:**
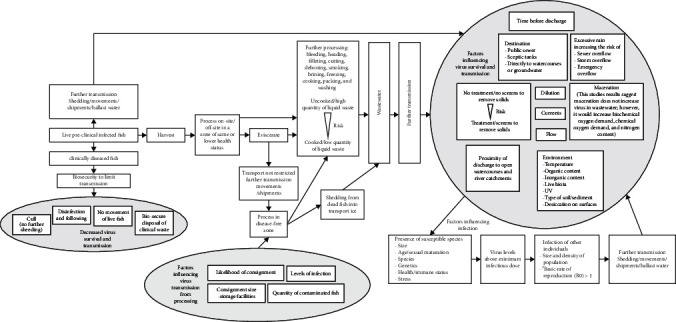
Transmission factors associated with processing pre-clinical infected fish. ^†^Basic rate of reproduction (R0) greater than one [54].

There are several factors that will influence the likelihood of pathogen introduction and onward spread to a previously pathogen-free area (summarised in Figure 5). Many of these risks have been assessed in qualitative risk assessments or disease investigations and must be taken into consideration [6, 10, 11, 14, 16, 43].

### 4.5. Likelihood of Processing Infected Fish

It is likely that most fish are processed within their country and less likely a VHSV-free country will receive an infected batch of fish for processing. There are currently more than 1,000 various fish processing facilities in the United Kingdom (https://data.food.gov.uk/catalog/datasets/1e61736a-2a1a-4c6a-b8b1-e45912ebc8e3, accessed 14 September 2021). Data from a survey in 2012 showed that in England and Wales, 34 out of just over 200 rainbow trout farms in the survey reported to process fish on-site, of which seven sourced fish from non-UK sources (13; Cefas, unpublished data). To date, there have only been suspected cases from the discharge from processing facilities, and it is unknown how many of these received fish from a high-risk zone or country.

### 4.6. Consignment Size of Fish

If receiving fish, the number and size will vary greatly depending on the capacity of the processing establishment and their facility to store fish prior to processing. However, if such consignments were imported, the size of the consignment could be substantial, and therefore, high quantities of virus arriving with it. This in turn influences the amount of virus discharged, as does the type of processing and volume of waste.

### 4.7. Type of Processing

Some processes inactivate viruses (such as cooking), but processing is often associated with generating considerable quantities of solid and liquid waste containing raw product, mucus, fat, oil, and grease with blood (as well as melting ice from transporting products—although there is no data on VHSV, there is evidence of infectious haematopoietic necrosis virus shedding from dead fish [43]). The volume of wastewater from fish processing is very diverse. Each processing facility is unique, so generalisations about water use and wastewater characteristics are difficult [44]. It is hard to obtain data from the industry for reference, but it is believed that the water volumes per weight unit of fish used in this study are realistic (250 mL of water per ca. 300 g)—if anything relatively high compared to the processed fish weight.

### 4.8. Wastewater Treatment

UK legislative requirements, intended to ensure adequate levels of wastewater treatment and protecting the water environment, are laid down in the Urban Waste Water Treatment Regulations 1994 and 1997 [45–47], while retained Regulation (EC) No 1069/2009 (Animal by-products Regulation) [48] sets out the requirements for animal by-products and derived products not, or no longer, intended for human consumption. Wastewater generated from processing animal by-products would be classed as a derived product and would thus be governed by the Regulation. The Regulation categorises animal by-products and derived products according to the level of risk to public and animal health. Wastewater treatment may further decrease viral levels and can involve primary treatment where by solids are removed by filters. This may then be followed by secondary or tertiary treatments. Secondary treatment involves the biological breakdown and reduction of residual organic matter. Anaerobic processes such as up flow anaerobic sludge blanket reactor, anaerobic filter, and anaerobic fluidised bed reactor can achieve high (80%–90%) organics removal and produce biogas. Aerobic processes such as activated sludge, rotating biological contactors, trickling filters, and lagoons are also suitable for organics removal. Anaerobic digestion followed by an aerobic process is an optimal process option for fish processing wastewater treatment [44]. Tertiary treatment involves chemical and physical forms of disinfection or the use of ultraviolet irradiation. Generally, primary screening in processing facilities may be carried out, but otherwise, liquid waste is often subject to minimal treatment [49]. Based on the viral levels recovered, this study shows that solid waste entering the open water may pose the highest risk, particularly in wastewater from eviscerating fish. During commercial processing, although large pieces of tissue (for example, the removed gut) are collected as solid waste, some pieces of tissues, such as blood, scales, and mucous, inevitably end up in the liquid waste. Once the solid fraction was removed, there was no significant difference in the levels found in the liquid waste between the processes.

Insufficient data are currently available to assess whether aquatic animal pathogens would be inactivated by the wastewater treatment applied to discharge from processing facilities at an acceptable level, but the Joint Government/Industry Working Group on Infectious Salmon Anaemia (ISA), whose membership comprised representatives from the Scottish Executive Environment and Rural Affairs Department, Fisheries Research Services, the State Veterinary Service, the Scottish Environment Protection Agency and the Scottish trout and salmon farming industries, concluded that one of the major risks associated with harvesting was environmental contamination with blood and other processing waste [15]. In particular, they recommended that in Scotland, blood water effluent from salmon processing plants should be disinfected before discharge, and other processing waste should be ensiled. Similarly, it has been found in Norway that farms within 5 km of a processing plant had an increased risk of the viral infection ISA, compared with farms more distant [50]; however, the disinfection treatment has been effective in reducing the number of ISA outbreaks in Norway [17, 39].

### 4.9. Maceration

Many processors will have systems in place to macerate solid waste released with their liquid waste prior to discharge. Blending the wastewater samples was intended to mimic the maceration processing and would be expected to cause the release of virus from the cells of the tissue into the liquid of the wastewater. There was, however, no statistical difference in the levels of VHSV in the liquid waste from blended or unblended samples after the blending and removal of solids.

The amount of solid waste separated from blended wastewater samples was lower than unblended samples, particularly for eviscerated fish wastewater, implying maceration reduces the solid waste without increasing the amount of virus in the liquid waste. Further replication experiments would be advised to confirm this, and the negative impact of increased organic waste should also be considered, such as increases in biochemical oxygen demand, chemical oxygen demand, and nitrogen levels. It is likely that blending of the kidney released nucleases and/or enzymes that decreased the viral titre overnight, or circulating neutralising antibodies are active in the homogenate, as has been indicated for infectious pancreatic necrosis virus in kidney homogenate [51]. Organic and inorganic content and live biota may also have inactivated some viruses in the 24 hr prior to titration [52]. This highlights that survival may be affected during the time from waste production to the time of discharge.

### 4.10. Location and Discharge Risks

The location of the processing facility, whether on- or off-farm-site, its proximity to pen water courses, river catchments, and the presence of susceptible specie scan affect transmission. Destinations of liquid waste include septic tanks, the public sewer, and directly to watercourses or groundwater. Discharge of liquid waste to a watercourse was judged as the route most likely to result in the infection of susceptible individuals [14]. Discharge of liquid waste to main sewerage requires the permission of the relevant water authority. However, discharge of untreated sewage through combined sewer overflows, storm overflows, or emergency overflows is a regular occurrence during periods of heavy rainfall in England and Wales, increasing the chance of discharge into the receiving water with a lack of efficient wastewater treatment. This may have limited or no disease implications if the processing units only source from their own site. However, processors can choose where to source fish from, and therefore, this risk may change considerably if such sites were processing fish from non-UK sources.

Once discharged, the dilution from discharge, flow rates, and currents all play a role in viral spread. Research on pathogens spread from infected farms via water currents suggests that neighbouring-naive farms can become infected via waterborne transmission [15, 50, 53].

### 4.11. Survival in the Environment

Throughout this process, the virus is also impacted by the environment. Active VHSV can persist for weeks depending on the environmental conditions (temperature, inorganic or organic content, live biota, UV light, adsorption to soil, and sediment) [12, 13, 52]. Survival has also been shown to last >56 days when VHSV was dried on stainless steel (often used for surfaces and equipment in processing facilities) and, when resuspended, may also enter the waste stream [52]. However, an outbreak will only occur if the virus, which has been released and survives in the aquatic environment, comes into contact with and infects a susceptible host(s) and that infected individual(s) infects more than one other individual (the basic rate of reproduction (R0) greater than one) [54].

Each aspect discussed above can cause a substantial variation in the epidemiology of the disease. Quantitative risk assessments and modelling would need to be undertaken to estimate the likelihood of the establishment of exotic pathogens more accurately in wild or farmed populations of aquatic animals.

## 5. Conclusions

This study provides quantitative data on the amount of VHSV initially shed from preclinical rainbow trout and the amount of VHSV in the tissues of these fish, leading to how much could be released when processing such fish. Our findings highlight that solid and liquid waste from processing can carry considerable virus loads, even when the animals processed are not showing clinical signs of infection. As fish can be transported from areas of low health status to declared disease-free areas if eviscerated, pathogen introduction is possible. While evisceration removes organs carrying a high pathogen load in infected fish, our results demonstrate that filleting of eviscerated fish imported from regions not declared free of VHSV, presents a potential route of pathogen introduction. Off-site processing may present a risk of pathogen transmission if susceptible fish are in close vicinity; therefore, biosecurity measures should reflect this.

## Figures and Tables

**Figure 2:**
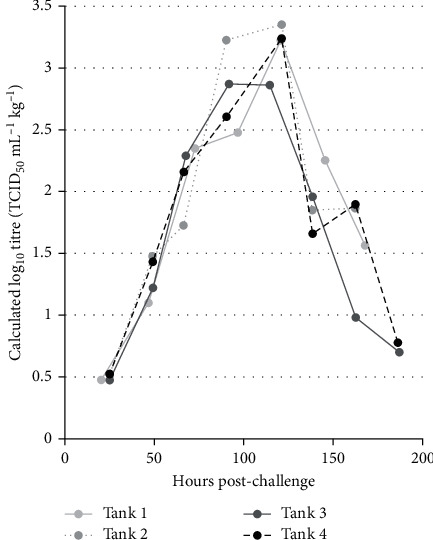
Shedding rate of VHSV J167 from rainbow trout post-challenge. The log_10_ titre of VHSV (TCID_50_ mL^−1^) shed kg^−1^ remaining fish was calculated at each sample point, from a mean titre of water samples from four areas of the tank, for four replicate exposure tanks. Tanks had continuous water flow (3 L min^−1^). The limit of detection (LOD) of the assay was 1.76 × 10^1^ TCID_50_ mL^−1^ (calculated to be 0.48 log_10_ TCID_50_ mL^−1^ kg^−1^ fish at the point of challenge—18 fish per tank).

## Data Availability

The data that support the findings of this study are available from the corresponding author upon reasonable request and in the supplementary material of this article.

## Supplementary Materials

Supplementary 1 Log_10_ titres of VHSV in pre-clinical fish tissues (eviscerated kidney and filleted muscle) and the solid and liquid fractions of centrifuged wastewater from eviscerating and filleting groups of four fish on day 6 or 8 post-challenge.

Supplementary 2 VHSV titres in kidney and muscle of moribund fish, mortalities and survivors at day 8 post-challenge and the internal clinical signs observed during dissection.
