# Supplementary material for: Shedding of Viral Haemorrhagic Septicaemia Virus (VHSV) from Rainbow Trout, Oncorhynchus mykiss, and Quantification in Waste from Processing Preclinical Fish
Source: Transbound Emerg Dis. 2023 Sep 28;2023:5534720. doi: 10.1155/2023/5534720 (PMC12017040; doi:10.1155/2023/5534720)
Supplement: Supplementary 1 — Log10 titres of VHSV in pre-clinical fish tissues (eviscerated kidney and filleted muscle) and the solid and liquid fractions of centrifuged wastewater from eviscerating and filleting groups of four fish on day 6 or 8 post-challenge. [file 5534720.f1.pdf]

Eviscerated or filleted	Sample day	Log_{10} titre in tissue ($TCID_{50}$ g^{-1})	Wastewater blended?	Log_{10} titre in solid fraction of wastewater ($TCID_{50}$ g^{-1})	Weight of solid fraction of wastewater (g)	Log_{10} titre in liquid fraction of wastewater ($TCID_{50}$ ml^{-1})	Log_{10} titre in liquid fraction of wastewater normalised ($TCID_{50}$ g^{-1} solid fraction)
Eviscerated	6	8.87	Yes	4.75	3.96	2.58	4.98
Eviscerated	6	10.25	Yes	8.75	2.17	6.87	9.53
Eviscerated	6	7.62	Yes	7.12	4.06	4.75	7.14
Eviscerated	6	8.91	No	8.49	13.15	1.41	3.29
Eviscerated	6	9.08	No	9.25	14.19	4.75	6.59
Eviscerated	8	6.87	Yes	5.99	2.74	4.08	6.64
Eviscerated	8	7.37	Yes	5.49	3.60	4.75	7.19
Eviscerated	8	8.75	Yes	7.87	3.53	5.91	8.36
Eviscerated	8	8.62	No	8.08	15.09	5.58	7.40
Eviscerated	8	7.49	No	7.37	11.85	3.41	5.34
Eviscerated	8	8.37	No	7.49	11.26	4.41	6.36
**Geomean**		8.21	Yes	6.52	3.34	4.61	7.16
**Geomean**		8.48	No	8.11	13.11	3.55	5.59
Filleted	6	5.49	Yes	6.25	2.02	4.75	7.44
Filleted	6	6.62	Yes	6.99	2.23	4.99	7.65
Filleted	6	5.25	Yes	5.87	4.68	3.75	6.08
Filleted	6	6.08	No	6.49	3.45	4.58	7.04
Filleted	6	6.75	No	7.12	6.23	4.91	7.12
Filleted	8	5.25	Yes	6.49	2.03	4.08	6.77
Filleted	8	4.62	Yes	6.25	2.61	3.91	6.49
Filleted	8	5.37	Yes	6.25	2.93	4.41	6.95
Filleted	8	5.25	No	6.25	3.27	4.58	7.06
Filleted	8	4.37	No	5.87	2.91	3.08	5.61
Filleted	8	5.12	No	6.37	4.21	3.91	6.29
**Geomean**		5.40	Yes	6.34	2.75	4.29	6.87
**Geomean**		5.45	No	6.41	4.01	4.16	6.60

Table S1: Log_{10} titres of VHSV in pre-clinical fish tissues (eviscerated kidney and filleted muscle) and the solid and liquid fractions of centrifuged wastewater from eviscerating and filleting groups of 4 fish, on day 6 or 8 post challenge. The normalised titre in the liquid fraction is the $TCID_{50}$ ml^{-1} in the total volume of liquid waste supernatant divided by the weight of the solid fraction (g).
