# Supplementary material for: Shedding of Viral Haemorrhagic Septicaemia Virus (VHSV) from Rainbow Trout, Oncorhynchus mykiss, and Quantification in Waste from Processing Preclinical Fish
Source: Transbound Emerg Dis. 2023 Sep 28;2023:5534720. doi: 10.1155/2023/5534720 (PMC12017040; doi:10.1155/2023/5534720)
Supplement: Supplementary 2 — VHSV titres in kidney and muscle of moribund fish, mortalities and survivors at day 8 post-challenge and the internal clinical signs observed during dissection. [file 5534720.f2.pdf]

Days post challenge	State	Internal signs of disease	Titre in muscle ($TCID_{50}\ g^{-1}$)
7	Near-Moribund	Severe haemorrhaging in the muscle near the head and fat surrounding the gut	5.56E+07
7	Near-Moribund	Mild haemorrhaging in the fat surrounding the gut. Very dark spleen and kidney. Moderate haemorrhaging in the muscle near the head with petechial haemorrhaging in the muscle further down the body	8.15E+07
7	Near-Moribund	Severe haemorrhaging in the fat surrounding the gut, Moderate haemorrhaging in the muscle, particualrly near the head	3.78E+07
6	Near-Moribund	No signs of disease	1.20E+08
7	Near-Moribund	Moderate haemorrhaging in the muscle	8.15E+07
7	Near-Moribund	No signs of disease	8.15E+05
8	Near-Moribund	Moderate haemorrhaging in the muscle	2.58E+07
8	Near-Moribund	Severe haemorrhaging in the muscle	3.78E+06
7	Near-Moribund	Ascites in the gut. Moderate haemorrhaging in the muscle	8.15E+07
8	Near-Moribund	Moderate haemorrhaging in the muscle near the head	5.56E+06
8	Mortality	Ascites and mild haemorrhaging in the muscle	3.78E+06
8	Mortality	Moderate haemorrhaging in the muscle	8.15E+05
7	Mortality	Severe haemorrhaging in the muscle and fat surrounding the gut	8.15E+07
8	Survivor	No signs of disease	3.78E+04
8	Survivor	No signs of disease	1.20E+05
8	Survivor	No signs of disease	2.58E+04
8	Survivor	No signs of disease	8.15E+04
8	Survivor	Moderate petechial haemorrhaging in the muscle	5.56E+05
8	Survivor	No signs of disease	1.20E+06
8	Survivor	No signs of disease	3.78E+06
8	Survivor	Moderate haemorrhaging in the muscle	8.15E+04
8	Survivor	Mild haemorrhaging in the muscle	8.15E+05
8	Survivor	No signs of disease	1.20E+06
8	Survivor	No signs of disease	3.78E+04
8	Survivor	No signs of disease	2.58E+04
8	Survivor	Moderate haemorrhaging in the muscle	5.56E+05
8	Survivor	No signs of disease	8.15E+03
8	Survivor	No signs of disease	1.20E+05

Table S2: VHSV titres in kidney and muscle of moribund fish, mortalities and survivors at day 8 post challenge internal clinical signs observed during dissection

Titre in kidney ($TCID_{50}$ g^{-1})
2.58E+10
8.15E+10
5.56E+10
1.20E+11
3.78E+10
5.56E+08
8.15E+08
2.58E+10
5.56E+10
1.20E+10
5.56E+09
5.56E+08
5.56E+10
1.20E+04
1.20E+06
2.58E+06
5.56E+03
1.20E+10
8.15E+06
5.56E+06
8.15E+08
1.76E+08
5.56E+07
3.78E+05
2.58E+06
5.56E+08
1.20E+08
1.20E+08

and the
